# Responses of the Necrotrophic Fungus *Alternaria brassisicola* to the Indolic Phytoalexin Brassinin

**DOI:** 10.3389/fpls.2020.611643

**Published:** 2021-01-14

**Authors:** Guillaume Quang N’Guyen, Roxane Raulo, Antoine Porquier, Beatrice Iacomi, Sandra Pelletier, Jean-Pierre Renou, Nelly Bataillé-Simoneau, Claire Campion, Bruno Hamon, Anthony Kwasiborski, Justine Colou, Abdelilah Benamar, Pietrick Hudhomme, David Macherel, Philippe Simoneau, Thomas Guillemette

**Affiliations:** ^1^UNIV Angers, Institut Agro, INRAE, IRHS, SFR 4207 QuaSaV, Angers, France; ^2^Institut Charles Viollette – EA 7394, Université de Lille, INRA, ISA, Université d’Artois, Université du Littoral Côte d’Opale, Lille, France; ^3^BIOGER – INRA UR1290, Thiverval-Grignon, France; ^4^USAMV, Bucharest, Romania; ^5^CNRS, Moltech Anjou, UNIV Angers, Angers, France

**Keywords:** phytoalexin, brassinin, fungus, necrotroph, mitochondria, signaling pathways, ergosterol

## Abstract

*Alternaria brassicicola* causes black spot disease in *Brassicaceae*. During host infection, this necrotrophic fungus is exposed to various antimicrobial compounds, such as the phytoalexin brassinin which is produced by many cultivated *Brassica* species. To investigate the cellular mechanisms by which this compound causes toxicity and the corresponding fungal adaptive strategies, we first analyzed fungal transcriptional responses to short-term exposure to brassinin and then used additional functional approaches. This study supports the hypothesis that indolic phytoalexin primarily targets mitochondrial functions in fungal cells. Indeed, we notably observed that phytoalexin treatment of *A. brassicicola* disrupted the mitochondrial membrane potential and resulted in a significant and rapid decrease in the oxygen consumption rates. Secondary effects, such as Reactive oxygen species production, changes in lipid and endoplasmic reticulum homeostasis were then found to be induced. Consequently, the fungus has to adapt its metabolism to protect itself against the toxic effects of these molecules, especially *via* the activation of high osmolarity glycerol and cell wall integrity signaling pathways and by induction of the unfolded protein response.

## Introduction

Plant antimicrobial secondary metabolites are one of the key elements of host immune systems. Two classes of plant antibiotics may be distinguished based on the type of synthesis in host cells (VanEtten et al., 1994). Phytoanticipins constitutively accumulate in plants or are produced during infection from preexisting constituents (Osbourn, 1996). On the contrary, phytoalexins are produced by plants in response to biotic or abiotic stresses, and accumulate to the infected site. The two types of molecules include a wide range of chemical families, but closely related plants generally synthesize phytoalexins of similar chemical structures. Phytoalexins have been demonstrated to have inhibitory activity *in vitro* against various bacteria and fungi, and they confered disease protection in several parasitic plant-host interactions (González-Lamothe et al., 2009; Ahuja et al., 2012; Großkinsky et al., 2012).

The *Brassicaceae* plant family, also known as crucifers, includes many plants of economic importance, among which many vegetables, ornamentals and oilseed. More than 40 phytoalexins have been identified from cultivated and wild *Brassicaceae* (Pedras et al., 2011). The majority contains an indole ring derived from (s)-tryptophan, and a sulfur atom derived from cysteine. However, non-indolyl cruciferous phytoalexins, such as nasturlexins A–D and corresponding sulfoxides, were recently reported in cress plants and appeared to be derived from the phenylalanine pathway (Pedras and To, 2015, 2018; Pedras et al., 2015). The most studied indolic phytoalexin is likely camalexin (3-thiazol-20-yl-indole) since this compound is the major phytoalexin produced by *Arabidopsis thaliana* (Ahuja et al., 2012). Brassinin [3-(S-methyldithiocarbamoyl) aminomethyl indole] is not present in this model plant species but is produced by many cultivated *Brassica* species. The compound is a precursor of several other phytoalexins, such as spirobrassinin and cyclobrassinin (Pedras et al., 2011). The toxophore group of brassinin is a dithiocarbamate group, which is present in broad-spectrum agrochemicals, and could be partly responsible for its antimicrobial activity.

Besides their antimicrobial activity against different plant pathogenic species, indolic phytoalexins are also known for exhibiting anti-trypanosomal activity (Mezencev et al., 2009), influencing cabbage aphid fitness (Kuśnierczyk et al., 2008), and having health-promoting effects. For instance, many of them have been found to contribute to antioxidant, anticarcinogenic and cardiovascular protective activities of *Brassica* vegetables (Jahangir et al., 2009). Recently, brassinin has been reported to have anti-proliferative effects against cancer through inhibition of the phosphatidylinositol 3-kinase signaling pathway (Izutani et al., 2012; Yang et al., 2019) and to suppress obesity-induced inflammatory responses (Kang et al., 2019).

Because of its antifungal activity (Sellam et al., 2007b), brassinin probably contributes to the level of resistance of plants against various fungal and oomycete pathogens, as it was well descibed for camalexin from studies on *Arabidopsis* mutants (Ferrari et al., 2003; Bohman et al., 2004; Van Baarlen et al., 2007; Chassot et al., 2008; Schlaeppi and Mauch, 2010; Schlaeppi et al., 2010). However, little information is currently available about the potential targets and cellular effects triggered by the exposure to brassinin. Sellam et al. (2007a) showed that camalexin probably damages fungal membranes and activates a compensatory mechanism in *Alternaria brassicicola* cells aimed at preserving membrane integrity. More recently, proteomic techniques were applied to detect differentially expressed proteins in *A. brassicicola* cultures exposed to camalexin or brassinin, respectively (Pedras and Minic, 2012; Pedras et al., 2014). The findings suggested that these respective phytoalexins affected protein expression differently. However, the exposure times that were applied in this study appeared too long to obtain relevant information on phytoalexin potential targets. We have reported earlier that an optimal adaptation of *A. brassicicola* to stress caused by camalexin and brassinin required the activation of several pathways: the unfolded protein response (UPR) and two mitogen-activated protein kinase (MAPK) signaling cascades, high osmolarity glycerol (HOG) and cell wall integrity (CWI) pathways (Joubert et al., 2011a, b). Other mechanisms, such as the production of detoxifying enzymes, can occur to protect cells against the toxicity of indolic phytoalexins. Some cruciferous pathogens are thus able to metabolize phytoalexins using a variety of reactions (Pedras et al., 2011). For example, *A. brassicicola* produces an inducible brassinin hydrolase, which transforms brassinin to 3-indolylmethanamine (Pedras et al., 2009). Srivastava et al. (2013) also reported that this fungus detoxified brassinin by transforming it into non-indolyl products. These authors highlighted the essential role of the *Bdtf1* transcription factor in the detoxification of brassinin and full virulence of the fungus.

*Alternaria brassicicola* is the causative agent of black spot disease in *Brassicaceae*. This ascomycetous fungus is a typical necrotroph that is exposed to several host indolic metabolites and that has to overcome their toxicity to achieve the infection process. In this study, we first focused on an analysis of the transcriptional response in germinating conidia to short-term exposures (0.5, 2, and 6 h) to brassinin at concentrations that would likely be found in the area surrounding necrotic lesions during host infection (Kliebenstein et al., 2005). The generated data and additional functional analyses provide insights on the cellular mechanisms by which the studied compound exerts its toxicity and on the strategies used by the fungus to protect itself against this defense metabolite.

## Results

### Susceptibility of *A. brassicicola* to Brassinin

**FIGURE 1:**
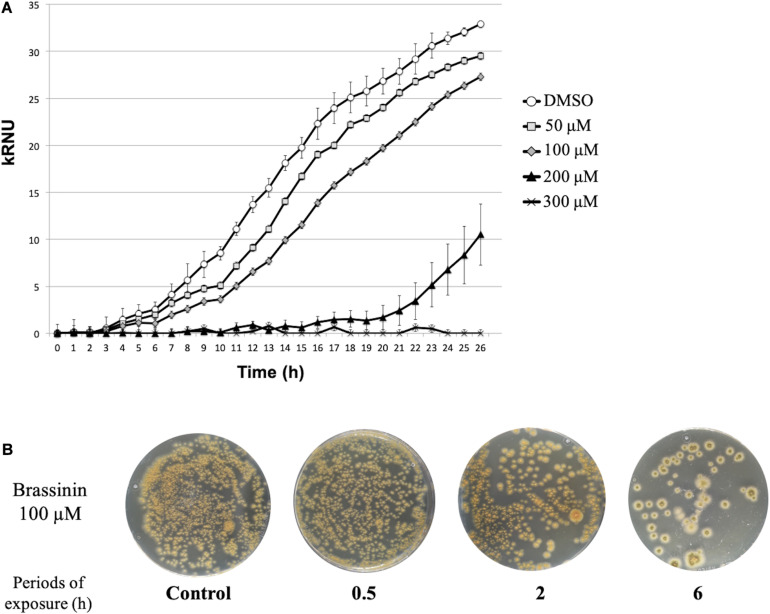
Susceptibility of *A. brassicicola* to brassinin. **(A)** Growth of the wild-type strain for 33 h at 24°C. The unit of the *Y*-axis corresponds to the relative nephelometric unit (RNU). Microplate wells containing standard PDB medium supplemented either with DMSO or various concentrations of brassinin were inoculated with a wild-type conidial suspension (10^5^ conidia/mL, final concentration). Fungal growth was recorded using a nephelometric reader. Each condition was conducted in triplicate and the experiments were repeated twice. Areas under the growth curves were used to calculate MGI_50_, i.e., the phytoalexin concentration for which 50% of mycelium growth inhibition was observed. **(B)** Effect of brassinin (100 μM) on viability of germinated *A. brassicicola* conidia. Conidia (10^5^ mL^– 1^) were germinated for 24 h, incubated in PDB for various times in the presence of brassinin at the desired concentration. After centrifugation, the pellets containing the germinated conidia were re-suspended in 200 μL, which were applied on the Petri dishes containing PDA medium. Colonies were visualized after 48 h of incubation. Control plates were prepared with conidia incubated for up to 24 h with DMSO (1% v/v final concentration).

We conducted several preliminary tests to define the optimal experimental conditions suitable for transcriptomic analyses (phytoalexin concentrations and sampling times). Phytoalexin sensitivity assays were first performed in 96-well plates by monitoring initial growth stages using laser nephelometry in liquid medium amended with different brassinin concentrations (Figure 1A).

For each tested condition, the fungal growth was assessed considering the area under the growth curve (AUC) as described by Gaucher et al. (2013). A percentage of growth inhibition was then calculated as follows [100-(AUC_*Brass*_/AUC_*c*_)^∗^100] by comparing the AUC obtained after exposure to brassinin to the AUC from the corresponding control culture. To avoid non-specific fungal responses to too severe stress conditions, we considered the MGI_50_ values (100 μM brassinin), corresponding to the phytoalexin concentration for which 50% of mycelium growth inhibition was observed, as the most suitable concentrations for studying the effect of brassinin. Moreover, this brassinin concentration corresponds to concentrations likely to exist in localized leaf areas surrounding necrotic lesions (Kliebenstein et al., 2005; Stefanato et al., 2009). Under these conditions and by using short exposure periods (0.5, 2, and 6 h), plating assays showed that cell viability did not differ from untreated controls (Figure 1B).

### Transcriptomic Analysis of the *A. brassicicola* Responses to Brassinin

**FIGURE 2:**
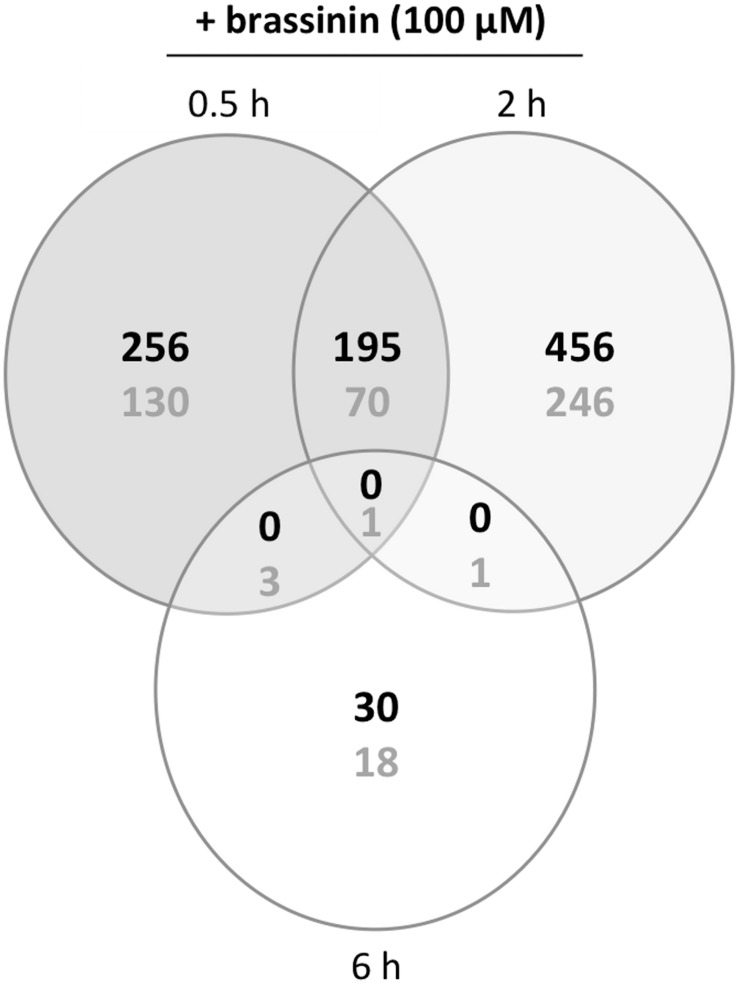
Venn diagram of overlapping and non-overlapping genes with significantly regulated expression levels in brassinin-treated cultures compared to DMSO-treated cultures. The number of induced or repressed genes are indicated with black or gray numbers, respectively. Probes with a *P* ≤ 0.01and a log ratio ≥ 1 or ≤-1 were considered as differentially expressed.

To explore cellular mechanisms by which the studied compound exerted its toxicity and further *A. brassicicola* protection mechanisms, we focused on the analysis of the transcriptional response in 1-day-old germinating conidia exposed to an MGI_50_ concentration of brassinin for 0.5, 2, and 6 h. At this stage, all conidia had produced short branched hyphae. Each phytoalexin-treated sample was compared to the control sample exposed to DMSO during an equivalent time. Thus, we produced three transcriptome datasets (each set was obtained from three biological replicates) using *A. brassicicola* microarrays. The numbers of genes differentially expressed under one or more conditions are shown in Figure 2. The expression profile of several genes following brassinin exposure was also confirmed by quantitative PCR (Supplementary Table 2).

First, our data showed that the number of differentially up-regulated genes in *A. brassicicola* cells following brassinin exposure was higher (almost twice) compared to the number of differentially down-regulated genes. Most genes appeared to be regulated by brassinin after 0.5 h, and especially after 2 h while very few were regulated after 6 h. The proportion of common genes up- or down-regulated at both time points (0.5 and 2 h exposures) was relatively low (around 20%), suggesting the occurrence of contrasting responses at each time point.

To gain insight into the *A. brassicicola* phytoalexin-related transcriptome, a Gene ontology (GO) enrichment analysis was performed to identify statistically over-represented GO terms within our given gene sets. No enriched GO terms were identified in the 6 h brassinin down-regulated gene list, probably because the number of genes was too low. Enriched categories emerging from the down-regulated genes after 0.5 and 2 h exposure were almost all related to the transcription and translation processes (Table 1). Within the up-regulated genes, the main enriched GO terms identified in the 0.5 and 2 h brassinin data sets were remarkably close and were predominantly associated with lipid metabolism and other related categories, such as actin cortical patch localization or vesicle-mediated transport and its child terms endocytosis and eisosome (Table 2). Almost all of these functional categories had obvious links to the cell membrane system, and the metabolisms of most of the membrane lipid families (phospholipids, sterols, and sphingolipids) were found to be over-represented within the data-sets. Distinct enriched categories emerged from the induced gene list obtained after 6 h of treatment. These categories were related to protein processing in endoplasmic reticulum (ER) and mitochondria, to cell wall biogenesis (chitosome) and to cellular transport (vesicle and myosin complex).

**TABLE 1:**
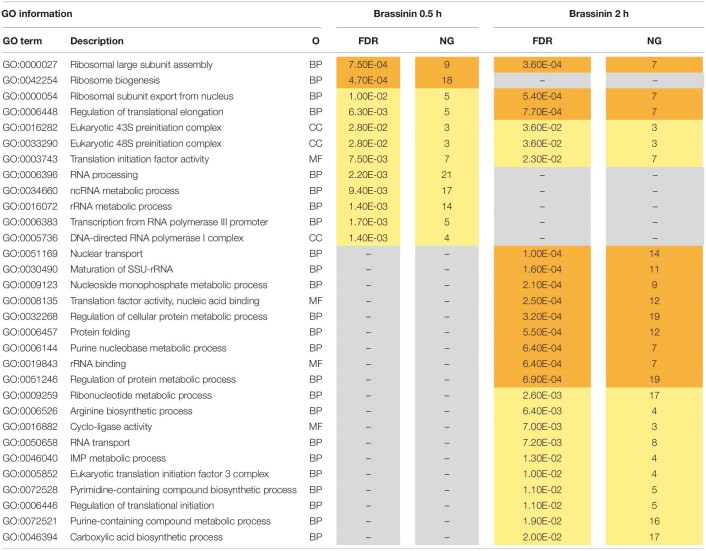
Gene ontology (GO) enrichment analysis on down-regulated genes related to brassinin exposure.

*The number of genes (NG) and P-values of the false discovery rate (FDR) are shown. O, ontologies; BP, biological process; CC, cellular component; and MF, molecular function.*

**TABLE 2:**
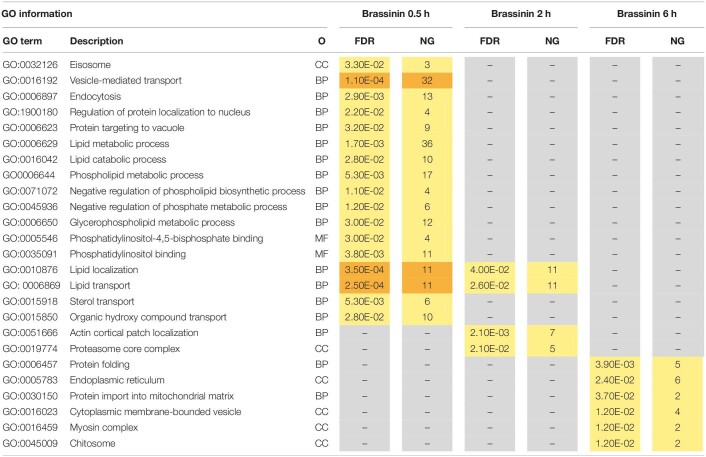
Gene ontology (GO) enrichment analysis on up-regulated genes related to brassinin exposure.

*The number of genes (NG) and *P*-values of the false discovery rate (FDR) are shown. O, ontologies; BP, biological process; CC, cellular component; and MF, molecular function.*

### Exposure to Brassinin-Induced Perturbations of Membrane Lipid Homeostasis

Our microarray analysis revealed that a number of mRNAs involved in the ergosterol pathway had increased abundance in samples treated by brassinin relative to DMSO-treated samples (Supplementary Table 3). These genes were directly related to ergosterol biosynthesis (such as the cytochrome P450 enzyme ERG5 and the transcriptional regulator UPC2), or to sterol transport (such as oxysterol-binding proteins). Sterols are essential lipid components of eukaryotic membranes and ergosterol is the major sterol in fungal membranes, modulating a wide variety of membrane processes, such as fluidity, permeability, and the activities of membrane-bound enzymes (Sturley, 2000). To test whether the ergosterol metabolism is linked to the fungal response to indolic metabolites, we generated two *AbErg5* deficient mutants using gene replacement cassettes and analyzed their response after exposure to brassinin. ERG5 is a sterol C-22 desaturase which is involved in the late steps of ergosterol biosynthesis. We chose to produce these particular mutants since the *AbErg5*-encoding gene was found to be over-expressed in two of the tested conditions (exposure to brassinin for 0.5 and 2 h, respectively; Supplementary Table 3).

**FIGURE 3:**
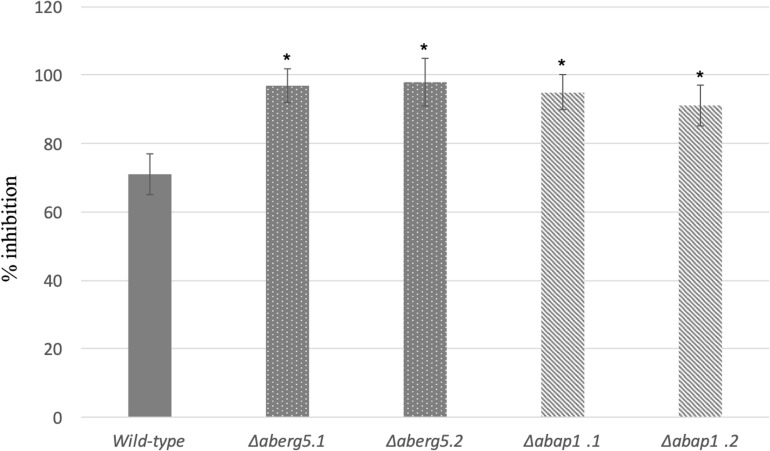
Growth inhibition rates of the wild-type strain and *AbErg5* and *AbAp1* deficient mutants (two transformants per genotype) exposed to 100 μM brassinin. The results show the percentages of inhibition in treated samples compared to the control. Fungal growth was recorded using a nephelometric reader from microplate wells containing standard PDB medium supplemented with brassinin or DMSO and inoculated with conidia (10^5^ mL^– 1^). Each genotype was analyzed in triplicate and the experiments were repeated three times per growth condition. Error bars indicate standard deviations. Asterisks indicate a significant difference between the mutant and the parental isolate (Student test, *P* < 0.01).

The susceptibility of the Δ*aberg5* strains to brassinin was assessed by nephelometric monitoring of the initial growth stages (Figure 3). Under control conditions (PDB medium), growth of the mutant strains was similar to that of the wild-type strain. However, the two mutants were found to be more susceptible than the wild-type to brassinin, thus supporting the fact that the sterol pathway plays a crucial role in the fungal response to this indolic metabolite.

### Brassinin Provokes Mitochondrial Dysfunction and Decreases Oxygen Consumption

Gene ontology enrichment analyses were conducted from the *A. brassicicola* automatically annotated genome database. In this database, the gene annotation was still partial and a large number of genes had no GO assignment, thus limiting the sensitivity of GO enrichment methods, such as GOEAST. A closer (manual) inspection of the up-regulated genes and their respective predicted function led us to identify other putative enriched functional categories in response to indolic phytoalexin treatment. First, a significant portion of genes (7%) transcriptionally induced by brassinin are involved in the oxidative stress response, such as glutathione transferases (GST), γ-glutamylcysteine synthetases, thioredoxins, thioredoxin reductase and oxidoreductases (Supplementary Table 4). Secondly, a large number of induced genes encode proteins which are predicted to be involved in mitochondrial functions or to be adressed to mitochondria (Supplementary Table 5).

**FIGURE 4:**
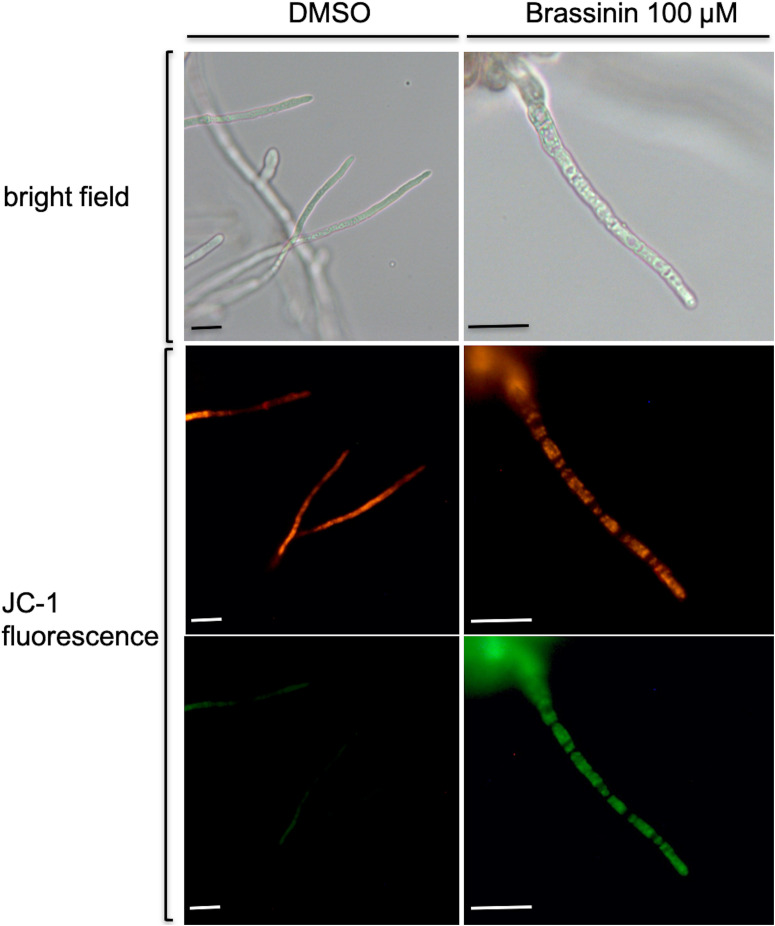
Assessment of changes in mitochondrial membrane potentials within *A. brassicicola* cells exposed for 30 min to 100 μM brassinin or to DMSO, using the fluorescent potentiometric dye JC-1. The top pictures correspond to light-field microscopy while the other pictures correspond to fluorescence microscopy. Scale bars = 20 μm.

As gene expression profiling revealed that a large group of mRNAs related to mitochondrial biogenesis and function showed an altered abundance following indolic phytoalexin treatment, we used the fluorescent cationic dye JC-1 to examine the status of the mitochondrial membrane potential in fungal cells exposed to brassinin. The JC-1 cationic dye accumulates in the mitochondrial matrix in a concentration-dependent manner. In healthy cells with a normal ΔΨ, JC-1 forms aggregates that display red fluorescence. In unhealthy cells with abnormal ΔΨ, the JC-1 dye also accumulates in the mitochondrial matrix but to a lesser concentration, not allowing the formation of JC-1 aggregates. In this case, JC-1 monomers display green fluorescence. The red/green fluorescence ratio of the dye is therefore an indicator of the mitochondria polarization and provide an assessement of the mitochondrial function. Compared to control (DMSO exposure), there was an increase in green fluorescence after exposure to brassinin, which was indicative of a decrease in mitochondrial ΔΨ (Figure 4).

**FIGURE 5:**
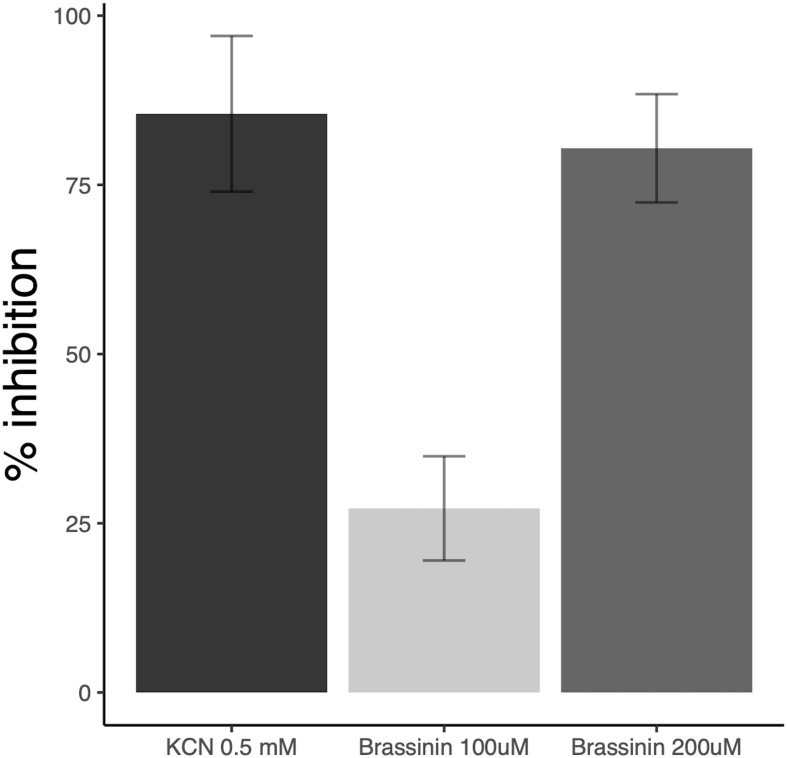
Impact of brassinin on *A. brassicicola* respiration. The results are expressed as the inhibitory effects (% of the control) on the respiration rate of germlings, following 1 h-exposure to 100 and 200 μM brassinin or 0.5 mM KCN. SD is indicated.

These alterations of ΔΨ indicate that brassinin can rapidly modify mitochondrial functions and processes related to cellular energy metabolism. The impact of brassinin on fungal respiration was then investigated by measuring the oxygen consumption of hyphae exposed to the phytoalexin for 60 min using a water soluble oxygen-sensitive fluorescent probe. Compared to the control, brassinin strongly inhibited the respiration of wild-type cells in a dose-dependent manner (100 and 200 μM were tested), reaching 80 % inhibition at 200 μM (Figure 5). By comparison, cyanide, the well-known and effective inhibitor of cytochrome c oxidase (component of mitochondrial electron transport), caused 85 % respiration inhibition at 500 μM.

Collectively, these results showed that mitochondria were a primary target of brassinin since it could affect both the mitochondrial membrane potential and oxygen consumption.

### Indolic Phytoalexin Induces an Intracellular Accumulation of Reactive Oxygen Species

**FIGURE 6:**
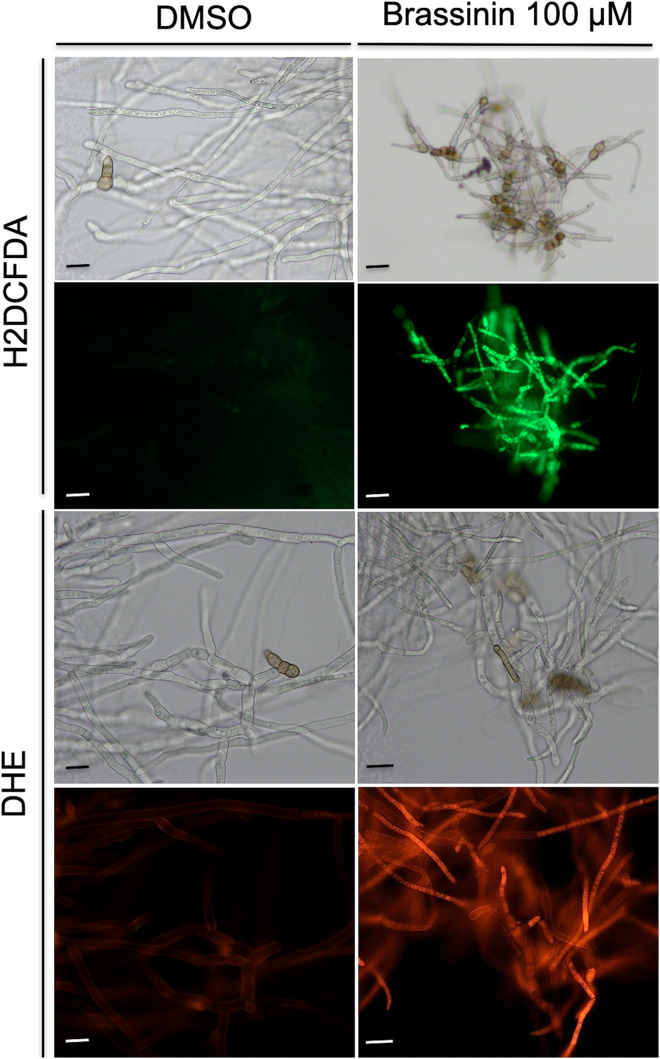
Assessment of oxidative stress within *A. brassicicola* cells exposed for 30 min to 100 μM brassinin or to DMSO. The fluorescent dyes H_2_DCFDA and DHE were used to detect the accumulation of ROS within hyphae of germlings treated for 1 h with DMSO (control) or 100 μM brassininin. For each panel, the bottom part corresponds to fluorescence microscopy and the top part to light-field microscopy. Scale bars = 20 μm.

To obtain additional support regarding brassinin-induced oxidative stress in fungal cells, Reactive oxygen species (ROS) accumulation was assessed using the fluorescent dyes 2’,7’-dichlorodihydrofluorescein diacetate (H_2_DCF-DA) and dihydro-ethidium (DHE). H_2_DCF-DA diffuses into cells and is hydrolyzed by intracellular esterases to form a nonfluorescent derivative. In the presence of intracellular ROS, this compound is rapidly converted to the highly green fluorescent 2’,7’-dichlorofluorescein (DCF). After 0.5 h or 1 h of incubation with brassinin, the amount of DCF-dependent fluorescence was increased in most of the examined hyphae, whereas low or no signal was recorded in the untreated control (Figure 6). We also used the fluorogenic dye DHE to detect intracellular superoxide or other ROS after exposure to brassinin. The level of DHE-derived fluorescence was increased in hyphae exposed for 0.5 h or 1 h to brassinin as compared with the untreated control (Figure 6), thus confirming that indolic phytoalexin induced intracellular ROS in fungal cells.

### Exposure to Brassinin Resulted in Activation of the MAP Kinase AbHOG1 and the Oxidant Responsive Factor AbAP1

Reactive oxygen species production in *A. brassicicola* following phytoalexin exposure induced the expression of various genes involved in antioxidant defenses. The expression of such genes was previously shown to be coordinated by several transcriptional regulators, either alone or in concert (de Dios et al., 2010; Morano et al., 2012). Our transcriptional data suggested that two regulators, i.e., the MAPK AbHOG1 and the AbAP1 transcription factor, might be preferentially involved in the response to phytoalexin-induced oxidative stress. For instance, several genes over-expressed following brassinin exposure were previously shown to be regulated by AbAP1, such as AB08663.1, AB00813.1, AB02955.1, AB04481.1 (Calmes et al., 2015), and *AbAp1* was up-regulated by brassinin. We also noticed the up-regulation of genes encoding putative osmosensors (such as AB03936.1 and AB09295.1) and other proteins able to interact with the HOG pathway (such as AB01195.1). The corresponding deficient single mutants Δ*abap1* and Δ*abhog1* were characterized by a high susceptibility toward oxidative stress generated by exposure to menadione (O_2_^–^ generation) or H_2_O_2_, as compared with their parental strain (Calmes et al., 2015).

**FIGURE 7:**
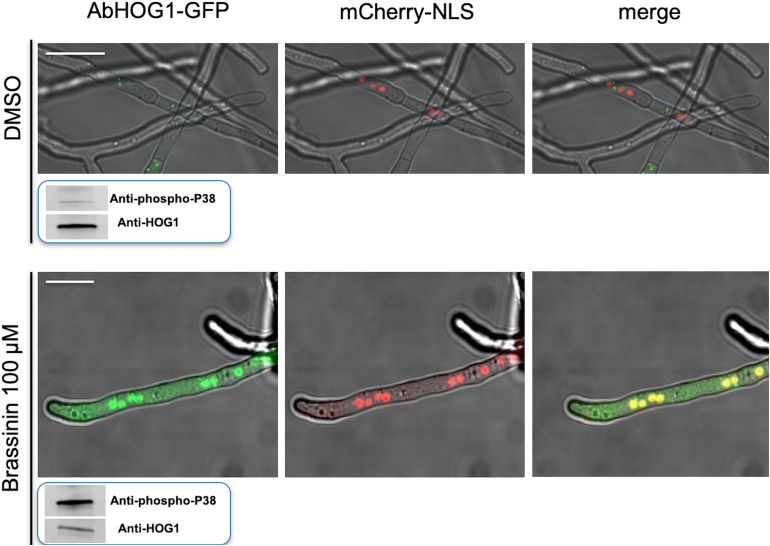
Cellular localization of the AbHOG1-GFP fusion protein in hyphae exposed to either DMSO (control) or 100 μM brassininin for 20 min and observed using confocal microscopy. Bars = 25 μm. A double-labeled strain expressing AbHOG1-GFP and mCherry-NLS was used. In smaller panels located under the pictures is presented the phosphorylation of the HOG1-like MAPK in *A. brassicicola* wild-type after exposure to brassinin. Total protein extracts were blotted with either anti-Hog1 *C*-terminus antibody or anti-dually phosphorylated p38 antibody.

In this study, we first determined the phosphorylation status of AbHOG1 in *A. brassicicola* wild-type when exposed to brassinin or DMSO for 20 min. We used a Western blot approach with antibodies specific of the phosphorylated form of p38-type kinases (Figure 7). Control blots were challenged with antibodies recognizing HOG1-type kinases in order to reflect the loading of the protein samples. As expected, increased phosphorylation of the AbHOG1 MAPK was observed upon challenge with brassinin, suggesting that AbHOG1 may play a crucial role in cell protection mechanisms. To confirm that AbHOG1 phosphorylation was followed by nuclear translocation, we investigated its subcellular localization during phytoalexin stress. AbHOG1-GFP fusion under the control of the *AbHog1* endogenous promoter was introduced in a *A. brassicicola* strain expressing a mCherry:NLS (nuclear localization signal) protein. This protein targets nuclei and allows their visualization in fungal cells (Khang et al., 2010). The mutant expressing the fusion protein was similar to *A. brassicicola* wild-type in terms of germination and vegetative growth on potato dextrose agar (PDA; data not shown). Under control conditions, GFP signals formed weak punctate dots localized in the cytoplasm (Figure 7). After a few minutes of treatment with brassinin, GFP-AbHog1 signals were found to be more intense and formed punctate green dots, which were localized in nuclei, as revealed by mCherry:NLS fluorescence.

**FIGURE 8:**
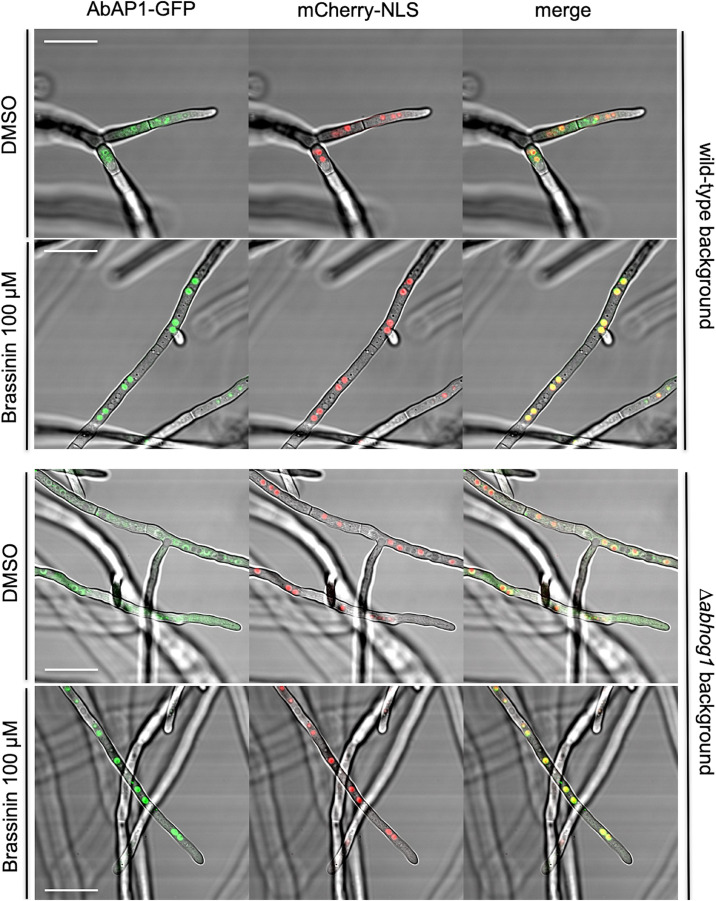
Cellular localization of the AbAP1-GFP fusion protein in the wild-type background and Δ*abhog1* background. Double-labeled strains expressing mCherry-NLS and AbAP1-GFP were exposed to either DMSO (control) or 100 μM brassininin for 0.5 h. Bars = 25 μm.

To assess the subcellular localization and a potential nuclear translocation of AbAP1 after exposure to brassinin, we constructed an *A. brassicicola* strain that expressed an AbAP1-GFP protein under control of the *Abap1* endogenous promoter and that also constitutively expressed the mCherry-NLS protein. After exposure of germlings to 100 μM brassinin for 20 min, we observed intense green fluorescent spots along the hyphae and their distribution co-localized with the red labeled nuclei (Figure 8). On the contrary, hyphal cells exposed to DMSO (control condition) exhibited more diffuse green fluorescence signals in the cytoplasmic compartment. A similar AbAP1 migration to nuclei was observed in an AbHOG1-deficient strain, indicating that AbAP1 was not under the control of the MAP kinase in response to brassinin (Figure 8).

When monitoring growth in solid PDA medium (data not shown) and in liquid PDB medium (Figure 3), Δ*abap1* strains did not show any growth defects and were not impaired in conidia germination and sporulation compared to the wild-type parental strain. However, as previously reported for the *AbHog1* mutants, increased sensitivities toward brassinin were recorded for Δ*abap1* strains compared to the wild-type (Figure 3).

In the light of the above results, we hypothesize that the HOG pathway and the AbAP1 transcription factor were activated by oxidative stress triggered by indolic phytoalexin exposure as a defense mechanism to cope with such stress.

### A Later Response to Brassinin Involved the UPR Activation

**FIGURE 9:**
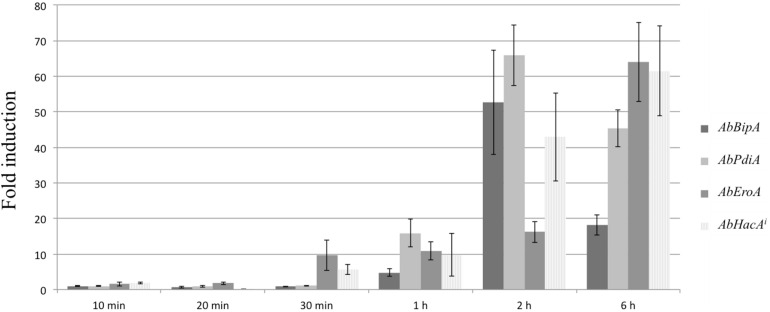
Brassinin activation of the UPR signaling pathway. Quantitative RT-PCR results for the expression of UPR target genes (*AbBipA*, *AbPdiA*, and *AbEroA*) and the spliced *AbHacA* mRNA (*AbHacA*^*i*^) in an *A. brassicicola* wild-type strain during brassinin exposure (200 μM) for 10, 20, and 30 min, 1, 2, and 6 h. For each target, expression induction is represented as a ratio (studied gene transcript abundance/actin transcript abundance) of its relative expression in each brassinin-treated sample to its relative expression in DMSO-treated cultures. The data are means of three repetitions. Error bars indicate standard deviations and asterisks indicate a relative expression significantly different from 1 (Student test, *P* < 0.01).

After 6 h of brassinin exposure, we observed the up-regulation of six genes (out of 30) which encode chaperone and protein disulfide isomerase, such as ER proteins AbPDIA (AB02021.1), which are essential for the formation of disulfide bonds in secretory and cell-surface proteins, and calnexin (AB10652.1) involved in folding and quality control of glycoproteins. Since these two proteins are known to be part of the UPR, we investigated whether brassinin was able to activate this pathway. UPR is a key element of ER protein quality control in eukaryotes allowing efficient maturation of membrane-bound and secreted proteins. In *A. brassicicola*, the basic leucine zipper (bZIP)-type transcription factor AbHACA is the major UPR transcriptional regulator (Joubert et al., 2011a; Guillemette et al., 2014). As initially described in the yeast *S. cerevisiae*, AbHACA synthesis is dependent on splicing of an unconventional intron in the *AbHacA* mRNA. In response to ER stress, this splicing event is promoted by the homolog of yeast ER-located transmembrane protein Ire1 (Rüegsegger et al., 2001; Joubert et al., 2011a) and triggers the translation of AbHACA, allowing the differential expression of UPR target genes. To confirm the activation of the UPR pathway in response to brassinin, we quantified the spliced *AbHacA* mRNA and monitored the parallel expression of three well-known UPR target genes, i.e., *AbPdiA*, *AbBipA* (encoding an ER chaperone), and *AbEroA* (thiol oxidase required for oxidative protein folding in the ER) in untreated and treated wild-type samples using quantitative RT-PCR (Figure 9). These analyses showed that *A. brassicicola* mycelium exposure to 200 μM of brassinin induced splicing of the *AbHacA* mRNA intron and also led to simultaneous up-regulation of the three target genes. UPR was activated after 1 h of treatment but induction was found to be stronger after 2 and 6 h exposure. In line with this, we previously showed that Δ*abhaca* mutants were hypersensitive to various host defense metabolites, including brassinin and camalexin (Joubert et al., 2011a), suggesting that UPR has a role in regulating a cellular compensatory response to preserve cell integrity during exposure to indolic phytoalexins.

## Discussion

While many studies have focused on structural diversity, chemical synthesis, biosynthesis, microbial transformations, and biological activities of cruciferous indolic phytoalexins (Pedras et al., 2011; Srivastava et al., 2013; Cho et al., 2014), the mechanisms by which these metabolites exert their toxicity on fungal cells have been so far poorly documented. Pedras and Minic (2012) and Pedras et al. (2014) compared protein profiles of *A. brassicicola* cultures treated for 20 h with either brassinin or camalexin. Overall, the findings of these studies highlighted clear differences in the cellular response to each phytoalexin and the authors suggested that each phytoalexin has several different targets in the cells. However, these studies were first conducted with the aim of identifying proteins potentially involved in the fungal protection from phytoalexin-induced stress. The exposure time that was applied thus seems to be too long and poorly suited to a strategy aiming at identifying cellular targets.

**FIGURE 10:**
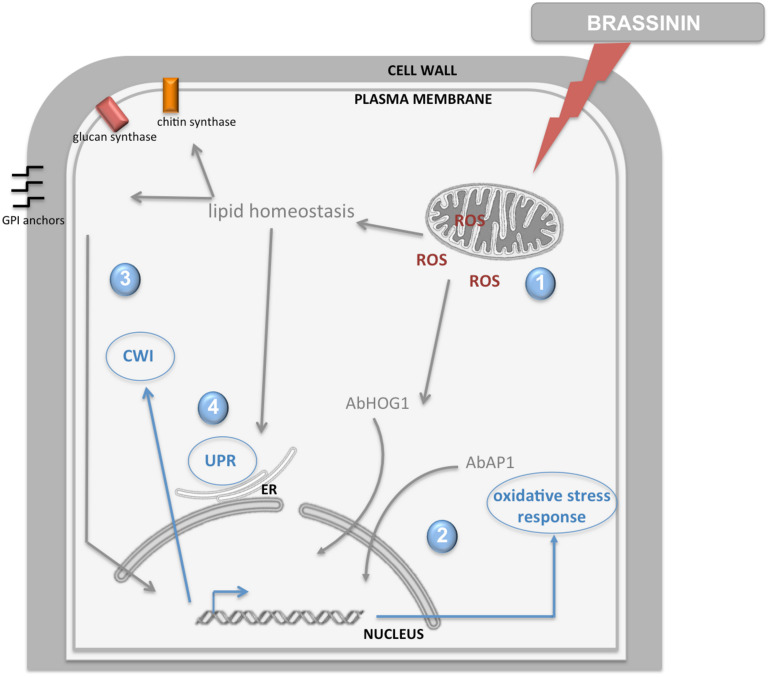
Model illustration of the fungal response induced by brassinin. The indolic phytoalexin primarily targets mitochondrial functions in fungal cells, inducing secondary effects such as ROS production and alteration of lipid homeostasis (1). Then, ROS production elicits AbHOG1 and AbAP1-mediated responses in order to reinforce antioxidant defenses and limit their deleterious effects (2). Changes in lipid homeostasis affect the activity of cell wall synthesis enzymes located in the plasma membrane and synthesis of the GPI anchor, leading to activation of the cell wall integrity pathway (3). Perturbations in membrane and cell wall homeostasis trigger UPR induction (4).

In the present study, young *A. brassicicola* cultures (germinating conidia) were exposed to brassinin for short times (0.5, 2, and 6 h) and the global transcriptional responses were compared. Overall, brassinin induced broad transcriptional regulation in fungal cells and the number of genes modulated by brassinin increased until at least 2 h of treatment and then decreased drastically after 6 h. Based on our transcriptional data and additional functional analyses, we propose an interpretative model showing the succession of cellular mechanisms that are activated after exposure to brassinin (Figure 10).

Our results supported the hypothesis that brassinin triggers mitochondrial dysfunction in *A. brassicicola* cells. First, a large group of mRNAs related to mitochondrial biogenesis and function showed an altered abundance following indolic phytoalexin treatment. Secondly, JC-1 dye revealed that phytoalexin treatment altered mitochondrial membrane potentials. Thirdly, exposure to brassinin (at concentrations corresponding to MGI_50_ or MGI_90_) resulted in a significant and rapid decrease in oxygen consumption rates of *A. brassicicola*. Our results thus suggested a probable effect on mitochondrial oxidative phosphorylation. Indeed, it is remarkable that several genes, whose expression is induced by phytoalexins, encode proteins involved in the respiratory chain, such as the NADH dehydrogenases NDE1 and NDE2 (complex I), lactate dehydrogenase and subunits of cytochrome c oxidase (complex III; Supplementary Table 5). As the reduction in oxygen consumption rates was found to be very fast (a few minutes after exposure to phytoalexin), we hypothesized that mitochondria are the primary target of brassinin. Another remarkable point is the simultaneous induction of several mitochondrial quality control pathways which are involved in maintaining mitochondrial integrity. Several genes encoding key antioxidant defense factors, such as thioredoxin and superoxide dismutase, are thus present in the list of induced genes and are known to detoxify mitochondrial ROS. Several other genes are involved in the mitochondrial protein quality control. Protein folding is indeed disturbed in case of excessive accumulation of ROS generated from the complex I and III of the electron transport chain (Hamanaka and Chandel, 2010; Tormos et al., 2011). In addition to molecular chaperones that are involved in promoting efficient mitochondrial protein folding and complex assembly, several genes encoding mitochondrial proteases (Lon proteases, metalloendopeptidases) were found to be induced by phytoalexin, probably to degrade proteins that failed to fold properly (Pellegrino et al., 2013). Another mechanism that emerged from the transcriptional data was the activation of the MAD (mitochondria-associated degradation) pathway. In this pathway, matrix or inner membrane proteins are retro-translocated to the outer membrane for ubiquitination and targeting to the proteasome (Chatenay-Lapointe and Shadel, 2010; Heo et al., 2010). Genes encoding the yeast homologous proteins Vms1p and Npl4p, which have been described as two key factors of MAD in yeast (Heo et al., 2010), were induced in *A. brassicicola* following phytoalexin exposure. All of these quality controls are important for clearing dysfunctional proteins from the inner membrane, which may accumulate due to failure in respiratory chain complex assembly or oxidative damage.

Mitochondrial alteration likely causes the generation of oxidative stress in fungal cells. Indeed, it is well described that mitochondrion appears to be the main intracellular source of ROS (Turrens, 2003) and, among the genes overexpressed by brassinin, a significant portion could be considered as related to an adaptive response to cellular oxidative stress (Supplementary Table 4). This observation suggested that the phytoalexins mediated redox dysregulation in fungal cells and ROS accumulation following phytoalexin exposure was revealed here using DHE and H2DFDA probes. We tried to use another intracellular ROS indicator, called MitoSOX Red, but, as previously reported by Calmes et al. (2015), this fluorescent compound is not suitable for *A. brassicicola* studies, probably due to its weak penetration through the fungal cell-walls. We also showed that ROS production elicited AbHOG1- and AbAP1-mediated responses (and possibly others) in order to reinforce antioxidant defenses and limit their deleterious effects.

In *A. brassicicola*, as in other filamentous ascomycetes, the HOG signaling pathway has a pivotal role in the response to hyper-osmotic and oxidant challenges (Hohmann, 2002; Moye-Rowley, 2003; Aguirre et al., 2005; Liu et al., 2008; Joubert et al., 2011b). This kind of stress triggers rapid phosphorylation and nuclear translocation of HOG1, the MAPK of the HOG pathway. In the nucleus, HOG1 associates with stress-responsive promoters and stimulates specific gene expression which is essential in the adaptive response of the cell (Hohmann, 2009; de Nadal and Posas, 2010). We previously showed that the HOG pathway has a pivotal role in regulating the protective fungal response during exposure to indolic phytoalexins (Joubert et al., 2011b). Camalexin and brassinin were found to phosphorylate AbHOG1 MAP kinase in a precocious manner and AbHOG1-deficient mutants showed hypersensitivity to these metabolites. Here we confirmed that brassinin exposure resulted in a rapid and transient accumulation of the AbHOG1 phosphorylated form in *A. brassicicola*. We also showed that the AbHOG1-GFP fusion protein migrated into the nucleus when fungal cells were challenged with brassinin, confirming the role of this MAP kinase as a tanscription factor under these conditions. It is likely that the nuclear AbHOG1 regulates the expression of a set of oxidative response genes.

Indeed, the AbHOG1-deficient mutant strain was not only found to be hypersensitive to indolic phytoalexins (Joubert et al., 2011b) but also to other oxidative stresses (Calmes et al., 2015). On the other hand, Alonso-Monge et al. (2009) showed that loss of HOG1 MAP kinase in *Candida albicans* resulted in an increased basal respiratory rate, an intracellular accumulation of ROS and a higher sensitivity to inhibitors of the respiratory chain. Similar crosstalks between the osmoregulatory pathway and respiratory metabolism could explain the high susceptibility of AbHOG1-deficient mutant to phytoalexins.

AP1-like transcription factors have also been shown to play major roles in the regulation of the oxidative stress responses in yeast and filamentous fungi (Lee et al., 1999; Aguirre et al., 2005). Yap1p, i.e., the yeast homologous protein of AbAP1, regulates the yeast peroxide detoxification pathway through activation by H_2_O_2_ and by inducing the transcription of most cellular antioxidants (Brombacher et al., 2006). Yap1p transcriptional activity is regulated by its cellular localization (Kuge et al., 1997; Gulshan et al., 2005). The *C*-terminal region contains a nuclear export signal (NES) and the *N*-terminal region has a NLS (Kuge et al., 1997). In the absence of oxidative stress, Yap1 is restricted to the cytoplasm by rapid nuclear export *via* exportin (Kuge et al., 1997; Yan et al., 1998). Upon exposure to stress signals, a conformational change leads to loss of the Yap1–Crm1 interaction, thus allowing Yap1p to accumulate in the nucleus (Gulshan et al., 2005). AP1-like transcription factors modulated the expression of various genes involved in antioxidant defenses, such as genes involved in thioredoxin and glutathione systems. For instance, overexpression of several GSTs and of the majority of the oxidative stress response genes in *A. brassicicola* cells challenged with isothiocyanates (ITCs) was found to be AP1-dependent (Calmes et al., 2015). Moreover, the AbAP1-deficient strain was found to be highly sensitive to oxidative stress caused by H_2_O_2_ menadione and ITCs (Calmes et al., 2015). In this study, we showed that this mutant strain had increased sensitivity toward brassinin compared to the wild-type. We also observed that the AbAP1-GFP fusion protein localized inside the nucleus upon exposure to phytoalexin. This result was indicative of cellular redox dysregulation triggering the conformational change and induction of AbAP1.

The increase in cellular ROS concentrations leads to free radical-mediated chain reactions, which target proteins, lipids, polysaccharides and DNA (Turrens, 2003; Aguirre et al., 2005). The damaging effects of ROS could explain some of the enriched GO categories that we identified from the transcriptome of phytoalexin-treated cells, such as categories linked to protein catabolism, transcription and translation processes and lipid metabolism. Our transcriptomic data clearly showed that the abundance of induced genes was related to various lipid pathways and membrane maintenance. In connection with this observation, it has been clearly shown that impairment of mitochondrial functions could have an effect on membrane lipid homeostasis. Indeed, the mitochondrial function is important for the membrane lipid structure, and thus, activation of lipid homeostasis could compensate for the aberrant membrane composition upon mitochondrial dysfunction (Shingu-Vazquez and Traven, 2011). Several observations support this point. Mitochondria are the predominant cellular site for synthesis of the ubiquitous membrane phospholipid phosphatidylethanolamine (PE; Schuiki et al., 2010), and PE also serves as the precursor for the synthesis of phosphatidylcholine, another major membrane phospholipid, in the ER (Birner and Daum, 2003; Schuiki et al., 2010). Hallstrom et al. (2001) also showed that sphingolipid homeostasis was disturbed in cells that have lost the mitochondrial genome. Moreover, heme acts as a cofactor for two cytochrome P450 enzymes in the biosynthetic pathway of ergosterol (including the sterol C-22 desaturase ERG5) and is synthesized in mitochondria (Crešnar and Petriè, 2011). In line with this, mitochondrial mutants in *C. albicans* and *C. glabrata* have an altered cellular sterol composition (Geraghty and Kavanagh, 2003; Brun et al., 2004). Moreover, Ichimura et al. (2000) showed that PE is required for the assembly of autophagosomes and delivery of cytoplasmic proteins to the vacuole by autophagy. We identified several genes related to autophagy that were over-expressed by brassinin (e.g., AB05971.1, AB06309.1, AB02770.1, AB02779.1, AB01785.1, and AB01424.1), and this induction may be linked to possible alteration of mitochondrial PE biosynthesis in phytoalexin-treated cells. MCC/eisosomes microdomains, that have been proposed to participate in the regulation of lipid homeostasis and also probably in the autophagic process, do not appear to be involved in the primary response to brassinin since Colou et al. (2019) showed that *A. brassicicola* mutants deficient for key MCC/eisosome components did not exhibit any enhanced susceptibility to this phytoalexin.

We previously reported that the CWI was required for adaptation of *A. brassicicola* to stress caused by camalexin and brassinin (Joubert et al., 2011b). Camalexin and brassinin were thus found to activate AbSLT2 MAP kinase and mutant strains lacking functional MAP kinase showed hypersensitivity to both phytoalexins. From our transcriptomic data, we also identified several genes related to cell wall synthesis, such as chitinase, glucanase, chitin synthase, mannoproteins and GPI (glycosylphosphatidylinositol)-anchored proteins (Supplementary Table 6). For instance, after 6 h of brassinin exposure, we reported up-regulation of six genes (out of 30) encoding proteins with functions in cell wall biogenesis and maintenance, and chitosome emerged as an enriched GO (cellular component) category (Table 1). Consistent with the mitochondrial targeting by brassinin that we report here, several studies recently revealed a link between mitochondrial dysfunction and CWI (Dagley et al., 2011; Shingu-Vazquez and Traven, 2011). Mitochondrial mutants of *C. albicans*, *Candida parapsilosis*, and *S. cerevisiae* were thus found to be sensitive to echinocandin antifungal drugs, which inhibit β-1,3 glucan synthase, the enzyme responsible for synthesis of the main glucan component of yeast cell wall (Chamilos et al., 2006; Sarinová et al., 2007; Hillenmeyer et al., 2008; Chen et al., 2010; Dagley et al., 2011). Moreover, it was reported that mitochondrial dysfunction leads to cell wall defects in *C. glabrata* and *C. albicans* (Brun et al., 2005; Batova et al., 2008; Dagley et al., 2011). Shingu-Vazquez and Traven (2011) put forward several hypotheses to explain how CWI may be affected by impairment of mitochondrial functions. Mitochondrial dysfunction could thus lead to changes in lipid homeostasis, which may affect the activity of cell wall synthesis enzymes located in the plasma membrane (such as chitin synthases). A second mechanism may affect the synthesis of the GPI anchor. A large number of cell wall proteins are GPI anchored, and PE, whose biosynthesis is dependent on mitochondria, is required for the synthesis of GPI structures. Finally, it is not really surprising that UPR was found to be secondarily activated by brassinin, since several studies showed that this pathway supports membrane and cell wall homeostasis (Malavazi et al., 2014). For instance, the cell wall composition is abnormal in Δ*hacA* and Δ*ireA* mutants (Richie et al., 2009; Feng et al., 2011; Guillemette et al., 2014), and both UPR mutants also showed decreased expression of mRNAs encoding enzymes in the ergosterol biosynthetic pathway and reduced total ergosterol levels (Richie et al., 2009; Feng et al., 2011). In addition, UPR mutants in *A. fumigatus* showed increased susceptibility to antifungal drug classes which target the plasma membrane and cell walls. Taken together, these results suggest that alterations in membrane and cell wall homeostasis due to brassinin activity may trigger UPR activation as an adaptive response. This is also strongly supported by the fact that loss of UPR in *A. brassicicola* Δ*abhaca* mutants resulted in an increased susceptibility to brassinin (Joubert et al., 2011a).

In conclusion, this study supports the hypothesis that indolic phytoalexin brassinin primarily targets mitochondrial functions in fungal cells, then inducing secondary effects such as ROS production and changes in lipid homeostasis. Consequently, the fungus has to adapt its metabolism to protect itself against the toxic effects of this molecule, especially via the activation of signaling pathways mediated by AbHOG1, AbAP1, AbHAC1, and AbSLT2. It is striking that there are several similarities between the responses to brassinin and mechanisms by which ITCs could trigger cell death in *A. brassicicola*. Many species of *Brassicaceae* constitutively accumulate secondary sulfur compounds called glucosinolates in relatively high levels. Glucosinolates and their breakdown products, including ITC, have significant antimicrobial activity against various plant pathogenic fungi. Calmes et al. (2015) showed that exposure of the fungus to ITCs resulted in a reduction of oxygen consumption, an intracellular accumulation of ROS and a mitochondrial-membrane depolarization. However, the effects on oxygen consumption were less pronounced after ITC exposure (40% inhibition of respiration at the MGI_90_ value) than after brassinin treatment (80% inhibition of respiration at the MGI_90_ value). Moreover, as shown in this study in response to brassinin, Calmes et al. (2015) found that AbHOG1 and AbAP1 were activated in the presence of ITCs and that the respective deficient mutants were hypersensitive to ITCs, suggesting a significant role of these regulators in fungal protection against ITCs. These results strongly suggest that common (at least in part) protection mechanisms could be induced by necrotrophic fungi against various brassicaceous defense metabolites. Developing specific inhibitors of said pathways may thus be a promising way to optimize the deleterious impacts of host defense metabolites and protect plants.

## Materials and Methods

### Antimicrobial Metabolites

Brassinin was synthesized according to Kutschy et al. (1998) and Takasugi et al. (1988) and dissolved in DMSO.

### Strains and Culture Conditions

The *A. brassicicola* wild-type strain *Abra43* (Sellam et al., 2007a) used in this study was grown and maintained on PDA at 24°C. Samples for transcriptomic analyses were prepared as described by Colou et al. (2019). Viability assays were performed at each time point by laying out aliquots of treated or control germlings onto PDA plates. The number and appearance of fungal colonies developed after 48 h of incubation revealed the impact of the treatment on the fungal viability.

Hyphal growth in liquid media were monitored over a 30 h period using a laser-based microplate nephelometer (NEPHELOstar, BMG Labtech) as described by Joubert et al. (2010). The fungal growth was assessed considering the AUC as described by Gaucher et al. (2013). Three replicates were conducted per treatment.

### RNA Extraction and Microarray Analysis

Total RNA extraction and microarray analysis were performed as described by N’Guyen et al. (2019) and Colou et al. (2019) using the dye-switch method on three biological replicates. Probes with *P* < 0.01 and with log ratio ≥ 1 or ≤−1 were considered as differentially expressed. Gene expression datasets were deposited in the Gene Expression Omnibus (GEO) with the following accession numbers, GSE140386.

### Generation of Targeted Gene Knockout Mutants and Fusion Strains

The gene replacement cassettes were generated using the double-joint PCR procedure (Yu et al., 2004) described by N’Guyen et al. (2019).

The AbHOG1 and AbAP1 *C*-terminal GFP fusion constructs were generated in the wild-type background by fusion PCR, as described in Calmes et al. (2015). Another AbAP1-GFP fusion mutant was also generated in the Δ*abhog1* background using the same procedure. Observations were performed under a Nikon (Nikon Instruments, Melville, NY, United States) A1S1 confocal laser microscope equipped with argon-ion (488 nm) and diode (561 nm) lasers.

### Western Blot Analysis

The phosphorylation status of HOG1-related MAPKs in *A. brassicicola* was studied as previously described by Calmes et al. (2015).

### Intracellular Detection of Oxidative Products

Assays with 2’,7’-dichlorodihydrofluorescein diacetate (H_2_DCF-DA, Molecular Probes) or dihydroethidium (DHE, Molecular Probes) solutions (5 μM final concentration) were perfoormed on young (16 h-old) germinating conidia exposed to either 100 μM brassinin or 1% (v/v) DMSO, as described by Calmes et al. (2015).

### Measurement of Mitochondrial Transmembrane Potential

Examination of the mitochondrial transmembrane potentials (ΔΨm) was performed as described by Calmes et al. (2015) using the dye 5,5′,6,′-tetrachloro 1,1′,3,3′tetraethylbenzimidazolylcarbocyanine iodide (JC-1; Invitrogen) on 16 h-old germinating conidia that were treated for the indicated times with either 100 μM brassinin or 1% (v/v) DMSO.

### Oxygen Consumption Rate Measurement

The respiratory activity was measured using the MitoXpress (Luxcel Biosciences, Cork, Ireland) fluorescent probe as previously descibed by Calmes et al. (2015).

### Quantitative PCR

Total RNA was extracted as described above. Amplification experiments were performed as described by N’Guyen et al. (2019) using the comparative ΔΔCt method (Winer et al., 1999). Primer sequences used in real-time quantitative PCR are summarized in Supplementary Table 1.

## Data Availability Statement

The datasets presented in this study can be found in online repositories. The names of the repository/repositories and accession number(s) can be found below: https://www.ncbi.nlm.nih.gov/geo/, GSE140386.

## Author Contributions

GN’G carried out sample preparation and microarray analyses. SP and J-PR were involved in gene expression analyses. PS and TG conceived the study, participated in the design of the experiments as well as in the analysis of the results. RR, AP, BI, CC, BH, and NB-S were involved in the construction and phenotyping of *A. brassicicola* mutant strains. JC performed assays for detection of oxidative products et measurement of mitochondrial membrane potential. DM and AB carried out tests to assess oxygen consumption rate. PH performed the brassinin synthesis.

## Conflict of Interest

The authors declare that the research was conducted in the absence of any commercial or financial relationships that could be construed as a potential conflict of interest.

## Supplementary Material

Supplementary Table 1 List of primers used in quantitative PCR.

Supplementary Table 2 Expression levels of genes (randomly selected from the microarray data) after brassinin exposure and estimated by quantitative PCR. Primer sets are indicated.

Supplementary Table 3 Genes induced by brassinin treatment and predicted to be involved in lipid metabolism. The indicated gene names correspond to homologous genes from the *Saccharomyces cerevisiae* genome except when indicated.

Supplementary Table 4 Genes induced by brassinin treatment and predicted to be involved in the oxidative stress response. The indicated gene names correspond to homologous genes from the *Saccharomyces cerevisiae* genome except when indicated.

Supplementary Table 5 Genes induced by brassinin treatment and predicted to be involved in mitochondrial functions. The indicated gene names correspond to homologous genes from the *Saccharomyces cerevisiae* genome except when indicated.

Supplementary Table 6 Genes induced by brassinin treatment and predicted to be involved in cell wall maintenance and biogenesis. The indicated gene names correspond to homologous genes from the *Saccharomyces cerevisiae* genome except when indicated.
